# CYP4X1 Expression Is Associated with Metastasis and Poor Prognosis in Patients with Colorectal Cancer

**DOI:** 10.3390/ijms26051867

**Published:** 2025-02-21

**Authors:** Sooyoun Kim, Hakchun Kim, Inpyo Hong, Minho Lee, Hyeongjoo Kim, Hyoungjong Kwak, Chang-Jin Kim, Donghyun Kang, Taesung Ahn, Moojun Baek, Dongjun Jeong

**Affiliations:** 1 Department of Pathology, College of Medicine, Soonchunhyang University, 31 Soonchunhyang 6-gil, Dongnam-gu, Cheonan 31151, Chungcheongnam-do, Republic of Korea; sooy.kim@sch.ac.kr (S.K.); hip0725@sch.ac.kr (I.H.); leemho00@sch.ac.kr (M.L.); 2 BK21 Four Project, College of Medicine, Soonchunhyang University, Cheonan 31151, Chungcheongnam-do, Republic of Korea; 3 Soonchunhyang Medical Science Research Institute, College of Medicine, Soonchunhyang University, 25 Bongjeong-ro, Dongnam-gu, Cheonan 31151, Chungcheongnam-do, Republic of Korea; khc5467@sch.ac.kr; 4 R&D Center Pharmaceutical Laboratory, Korean Drug Co., Ltd., 34, Nonhyeon-ro 28-gil, Gangnam-gu, Seoul 06300, Republic of Korea; pmbaki2@gmail.com; 5 Research Institute of Clinical Medicine, Woori Madi Medical Center, 111 Baekjedae-ro, Wansan-gu, Jeonju 55082, Jeollabuk-do, Republic of Korea; k-h-jong@hanmail.net (H.K.); mountain48@hanmail.net (C.-J.K.); 6 Department of Surgery, College of Medicine, Soonchunhyang University, 31 Soonchunhyang 6-gil, Dongnam-gu, Cheonan 31151, Chungcheongnam-do, Republic of Korea; c100048@schmc.ac.kr (D.K.); eyetoeye@schmc.ac.kr (T.A.); ssurge@sch.ac.kr (M.B.)

**Keywords:** *CYP4X1*, colorectal cancer, biomarker, prognostic marker, therapeutic target

## Abstract

Globally, the mortality rate of colorectal cancer (CRC) remains high. Despite the development of various treatments, such as targeted therapy and immunotherapy, colorectal cancer continues to be a serious health issue worldwide. Identifying new biomarkers is essential for improving prognosis and tailoring targeted therapies for CRC. This study aims to elucidate the role of *CYP4X1* in CRC and its association with patient survival and clinicopathological parameters. Using TCGA databases like GENT2, UALCAN, and GEPIA, we analyzed *CYP4X1* expression in CRC and normal tissues. Our analysis revealed a significant increase in *CYP4X1* expression in CRC tissues compared to normal tissues. And *CYP4X1* high expression was strongly associated with advanced TNM stage, poor tumor differentiation, deeper invasion, and lymph node metastasis. Kaplan–Meier analysis revealed that high *CYP4X1* expression correlated with shorter survival times. To investigate the relationship between *CYP4X1* expression and colon cancer, WST-1, Transwell, and colony formation assays were performed using colon cancer cells with siRNA-mediated *CYP4X1* downregulation. *CYP4X1* downregulation significantly inhibited cell proliferation, invasion, migration, and colony formation in vitro. Furthermore, the tumor-forming ability in mice injected with cell lines where *CYP4X1* expression was suppressed decreased. In conclusion, *CYP4X1* overexpression is closely linked to CRC progression as an independent prognostic marker and potential therapeutic target.

## 1. Introduction

Cancer is the most common cause of death worldwide. Colorectal cancer (CRC) is the third most common malignant tumor and the second most common cause of cancer-related deaths worldwide [1]. In addition, the relapse rate of CRC after resection is 30–50%, and half of patients with CRC develop metastasis [2,3]. In the United States, the 5-year relative survival rate of patients with CRC ranges from 90% in patients with confined disease to 14% in patients with distant metastasis [4]. Predicting recurrence and metastasis is crucial for the efficient treatment of patients with CRC. However, despite numerous studies, biomarkers for predicting CRC recurrence and metastasis have yet to be identified.

Cytochrome P450 (CYP4) enzymes constitute a superfamily predominantly localized to the inner mitochondrial membrane or the endoplasmic reticulum membrane in eukaryotic cells. To date, more than 11 CYP4 subfamilies have been identified, with six gene subfamilies reported: *CYP4A*, *CYP4B*, *CYP4F*, *CYP4V*, *CYP4X*, and *CYP4Z* [5]. The CYP4 enzyme family plays a pivotal role in the oxidation of various endogenous and exogenous compounds. These enzymes are inducible and are critically involved in lipid metabolism, thereby indirectly influencing drug responses in pathological conditions such as fatty liver disease, inflammatory disorders, and diabetes [6,7]. In addition, the CYP4 enzyme family plays a crucial role in tumorigenesis due to its involvement in the metabolism of various carcinogens [8]. Carcinogens implicated in the etiology of colorectal cancer (CRC) include polycyclic aromatic hydrocarbons, particularly heterocyclic amines, many of which undergo metabolic activation by CYP4 enzymes before exerting their genotoxic effects [9,10]. Abnormal CYP4 expression in cancer cells affects cell proliferation, signaling, and drug metabolism [11]. Approximately a quarter of CYP4 enzymes are considered orphans because their expression patterns, regulation, and functional information remain unknown [12,13,14].

Cytochrome P450 family 4, subfamily X, polypeptide 1 (*CYP4X1*) is a member of thecytochrome P450 superfamily. Human *CYP4X1* is classified as an orphan CYP, owing to its unknown functions. Human *CYP4X1* expression is mainly seen in the aorta, trachea, and skeletal muscles in adults [15]. Although the metabolic capacity of *CYP4X1* is unknown, a recent study has confirmed that arachidonic acid derivatives are involved in several essential physiological processes [16]. The arachidonic acid derivative arachidonoyl ethanol amide (anandamide) is a natural endocannabinoid found in human tissues that functions as a vital signaling mediator in immune, neuronal, and cardiovascular functions [16]. *CYP4X1* plays a central role in neurovascular brain functions [17] and has essential roles in several cancers. *CYP4X1* is associated with cancer activity [17,18,19,20]; its overexpression has been reported in breast [19], gastric [20], and brain cancers [17]. Thus, human *CYP4X1* is a potential drug target for cancer therapy. However, the role of *CYP4X1* in CRC remains unclear. The aim of this study is to investigate the functional role of *CYP4X1* expression in cancer, both in vitro and in vivo, and to correlate *CYP4X1* expression with clinical parameters in cancer patients. By doing so, we aim to propose *CYP4X1* as a potential prognostic biomarker in CRC and offer innovative targeted strategies for treatment.

## 2. Results

### 2.1. CYP4X1 Expression Patterns in Diverse Cancer and Normal Types: A Comparative Analysis Using GENT2, UALCAN, and GEPIA

**Figure 1:**
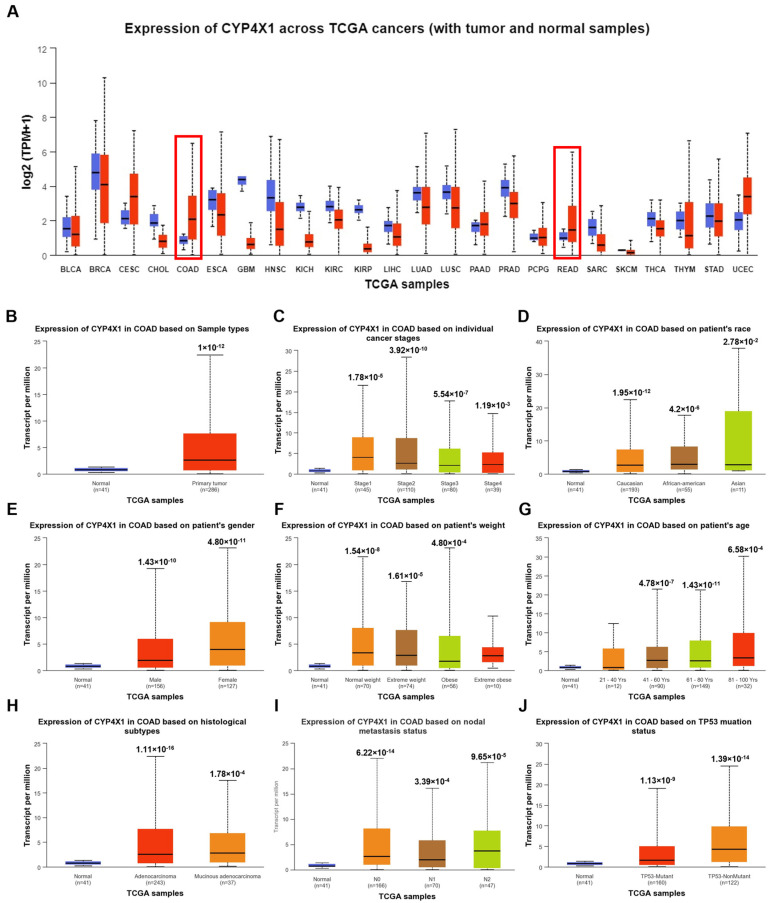
Analysis of *CYP4X1* mRNA expression by cancer type and variable using the UALCAN database. (**A**) *CYP4X1* mRNA expression levels in normal and tumor tissues across diverse cancer types were obtained from the UALCAN database. Red indicates the boxplot for cancer samples, while blue indicates the boxplot for normal samples. CYP4X1 expression is higher in colon (COAD) and rectal (READ) adenocarcinoma tissues compared to normal tissues, as indicated by the red boxes. Analysis of mRNA expression of *CYP4X1* based on multiple variables in (**B**) colon adenocarcinoma (COAD), (**C**) individual cancer stage, and the patient’s (**D**) race, (**E**) gender, (**F**) weight, (**G**) age, (**H**) histological subtype, (**I**) nodal metastasis status, and (**J**) TP53 mutation status.

Using TCGA data, the GENT2, UALCAN, and GEPIA databases were employed to compare *CYP4X1* expression in various cancer types and normal tissues. As a result, a higher expression of *CYP4X1* was observed in normal tissues compared to cancer tissues in esophageal carcinoma (ELCA), glioblastoma multiforme (GBM), head and neck squamous cell carcinoma (HNSC), kidney cancer (KIRC; kidney renal clear cell carcinoma, KIRP; kidney renal papillary cell carcinoma), and thyroid carcinoma (THCA). However, in rectal adenocarcinoma (READ), colon adenocarcinoma (COAD), and endometrial and cervical cancers (CESC; cervical squamous cell carcinoma and endocervical adenocarcinoma, UCEC; Uterine Corpus Endometrial Carcinoma), higher expressions of *CYP4X1* was observed in cancer tissues compared to normal tissues (Figure 1A). Based on various clinicopathological parameters, we analyzed the mRNA expression patterns of *CYP4X1* in normal rectal tissues and colon adenocarcinoma (COAD). Statistical analysis of the UALCAN database data revealed that *CYP4X1* expression was significantly higher in colon cancer tissues compared to normal tissues (*p* < 1 × 10^−12^; Figure 1B). Moreover, *CYP4X1* expression was elevated in stage 4 (distant metastasis) compared to normal tissues (*p* < 1.20 × 10^−3^; Figure 1C). Additionally, the correlation of *CYP4X1* expression with various clinicopathological factors, including sample type, individual cancer stage, race, weight, gender, age, histological subtype, nodal metastasis, and TP53 mutation status, showed higher expression in all COAD tissues compared to normal tissues (Figure 1B–J, Table 1).

### 2.2. High Expression of CYP4X1 Is Associated with Poor Prognosis in Patients with Colorectal Cancer

**Figure 2:**
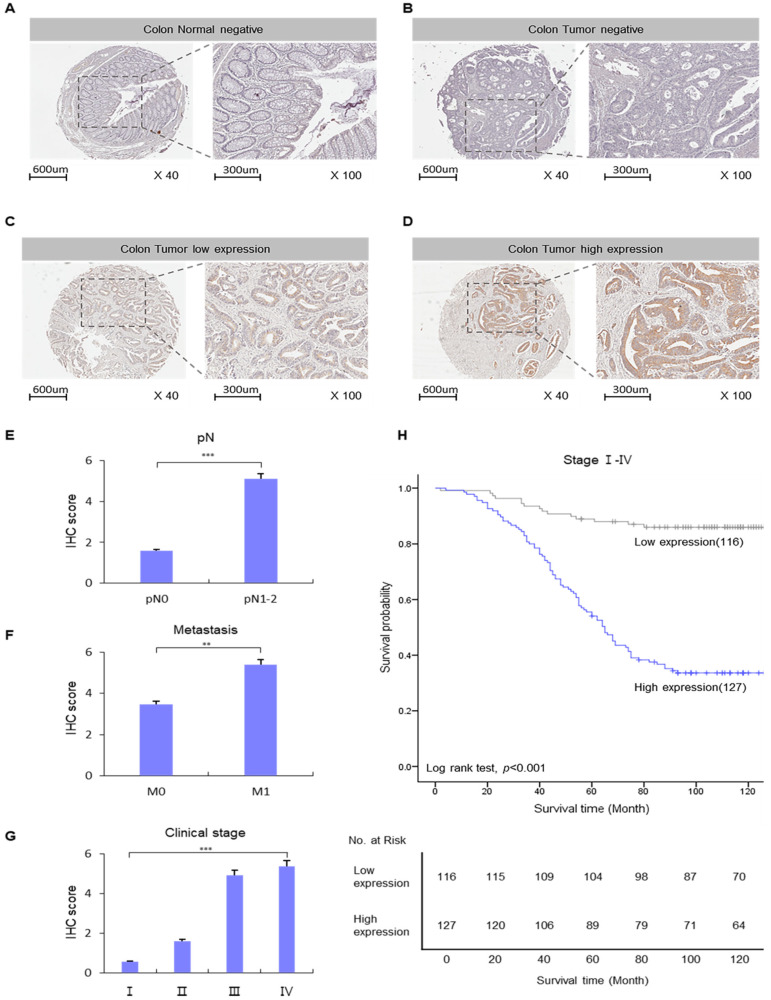
Correlation between *CYP4X1* expression and survival rate of patients with colorectal cancer (CRC). *CYP4X1* expression was confirmed in CRC tissues using immunohistochemistry (IHC) staining: (**A**) normal negative, (**B**) tumor negative (**C**), tumor low expression, (**D**) tumor high expression. IHC scores of *CYP4X1* expression according to the clinicopathology factor were evaluated using Mann–Whitney and Kruskal–Wallis tests. (**E**) pN, (**F**) metastasis, and (**G**) clinical stage (** *p <* 0.01, *** *p* < 0.001). (**H**) *CYP4X1* expression was associated with the 5-year overall survival of patients with CRC.

The expression of *CYP4X1* was compared and analyzed using IHC staining of normal colon tissues (adjacent to cancer) and CRC tissues obtained from 243 patients with CRC. *CYP4X1* expression showed high levels of over-staining primarily on the cell membrane, while low expression was observed in the cytoplasm. Furthermore, *CYP4X1* expression was higher in CRC than in normal colon tissues (Figure 2A). Low *CYP4X1* expression was observed in 116 of the 243 CRC tissues analyzed, while high expression levels were detected in 127 tissues (Table 2). *CYP4X1* overexpression significantly correlated with pN (*p* < 0.001, Figure 2B, Table 3), distant metastasis (*p* = 0.004, Figure 2C, Table 3), and clinical stage (*p* < 0.001, Figure 2D, Table 3). The clinical relationship between *CYP4X1* expression and patients with CRC was also investigated. A univariate analysis indicated that the following factors were specifically related to overall survival: pN: 11.833 (5.959–23.497), *p* < 0.001; distant metastasis: 3.949 (2.364–6.597), *p* < 0.001; clinical stage: 16.627 (7.277–37.988), *p* < 0.001; and *CYP4X1* expression: 8.068 (4.655–13.985), *p* < 0.001. A multivariate analysis also demonstrated that the following factors were significantly associated with overall survival: distant metastasis, 4.097 (2.282–7.355), *p* < 0.001; and *CYP4X1* expression, 3.532 (1.813–6.880), *p* < 0.001 (Table 4). Kaplan–Meier analysis comparing the survival rates of colorectal cancer patients based on *CYP4X1* expression revealed that patients with high *CYP4X1* expression had shorter survival times than those with low *CYP4X1* expression (log-rank test, *p* < 0.001; Figure 2E). These results demonstrate a correlation between *CYP4X1* overexpression and poor prognosis in CRC patients, showing a strong association with distant metastasis in colorectal cancer patients.

### 2.3. Inhibitory Effect of siRNA on CYP4X1 mRNA and Protein Expression in CRC Cell Lines

**Figure 3:**
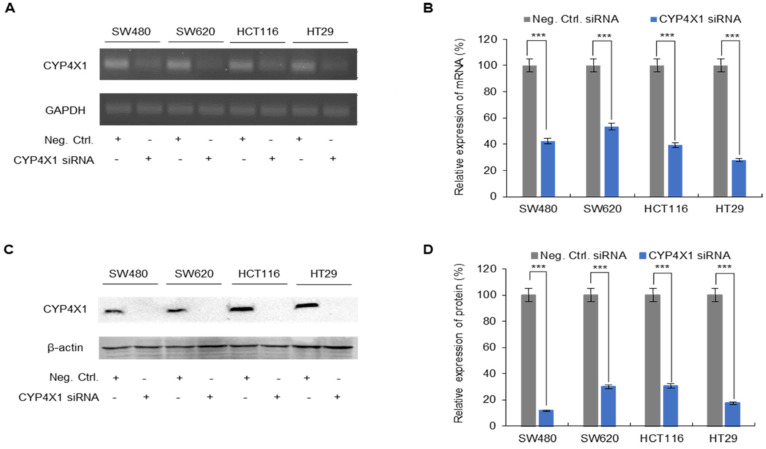
*CYP4X1* inhibition in human CRC cells using siRNA was confirmed using RT-PCR and immunoblotting. (**A**) Gel electrophoresis of PCR products shows the mRNA levels of the negative control compared to that of the *CYP4X1*-knockdown cell lines. (**B**) Percentage inhibition of *CYP4X1* expression in the *CYP4X1*-siRNA-treated samples compared to that in negative control. (**C**) *CYP4X1* protein knockdown was confirmed using immunoblotting analysis. (**D**) Relative protein expression of *CYP4X1* in the siRNA-treated samples compared to the negative control (*** *p* < 0.001).

siRNA was used to explore the role of *CYP4X1* expression in CRC cell function. *CYP4X1* mRNA expression was significantly downregulated in all colorectal cancer cell lines (*p* < 0.001; Figure 3A,B). The average *CYP4X1* expression in the *CYP4X1* siRNA-treated cell group was reduced by 59.3 ± 3.7% compared to that in the negative control cell group (SW480; 57.5 ± 6.2%, SW620; 46.8 ± 3.3%, HCT116; 60.8 ± 2.4%, and HT29; 72.1 ± 3.2%). In addition, Western blot analysis indicated a decrease in the protein level of *CYP4X1* expression in CRC cells (*p* < 0.001; Figure 3C,D). Compared to the negative control group of each cell line, the protein expression levels of the *CYP4X1* siRNA-treated group decreased as follows: SW480, 88.3 ± 7.3%; SW620, 69.9 ± 4.4%; HCT116, 69.4 ± 6.6%; and HT29, 82.6 ± 11.7%. Cells in which efficient *CYP4X1* inhibition was confirmed were used for functional studies regarding cell proliferation, migration, invasion, and colony formation.

### 2.4. CYP4X1 Expression Was Associated with Human CRC Cell Proliferation

**Figure 4:**
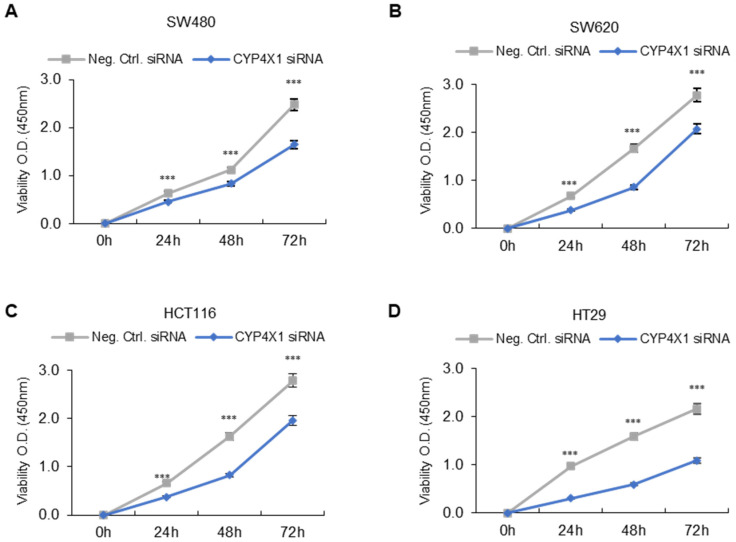
Inhibition of *CYP4X1* decreased the proliferative ability of colorectal cancer (CRC) cells. Cell proliferation of the siRNA-treated negative control and *CYP4X1* siRNA-treated cell groups represents the average of the 450nm absorbance measurements over time. Reduced cell proliferation was observed in the *CYP4X1* siRNA-treated cell group compared to the siRNA-treated negative control group. (**A**) SW480, (**B**) SW620, (**C**) HCT116, and (**D**) HT29 cells (*** *p* < 0.001).

The WST-1 assay was performed to investigate the association between *CYP4X1* expression and the proliferation patterns of human CRC cells. The proliferation of negative control and *CYP4X1* siRNA-treated cells was compared. CRC cells with downregulated *CYP4X1* expression showed reduced proliferation compared to the negative controls. The inhibitory effect on cell proliferation was significant at 24, 48, and 72 h after transfection (*p* < 0.001; Figure 4A–D). The proliferation rate of the *CYP4X1* siRNA-treated groups compared to the negative control groups decreased as follows—at 24 h: SW480, 23.31 ± 7.5%; SW620, 47.81 ± 4.4%; HCT116, 43.17 ± 4.6%; and HT29, 67.32 ± 5.6%—at 48 h: SW480, 19.03 ± 3.2%; SW620, 46.21 ± 4.6%; HCT116, 47.15 ± 3.8%; and HT29, 62.09 ± 6.3%—and at 72 h: SW480, 32.64 ± 4.2%; SW620, 29.38 ± 7.0%; HCT116, 31.46 ± 2.2%; and HT29, 51.42 ± 6.6%. These results demonstrate that *CYP4X1* inhibition inhibits the proliferation of CRC cell lines.

### 2.5. CYP4X1 Downregulation Affects CRC Cell Migration and Invasion

**Figure 5:**
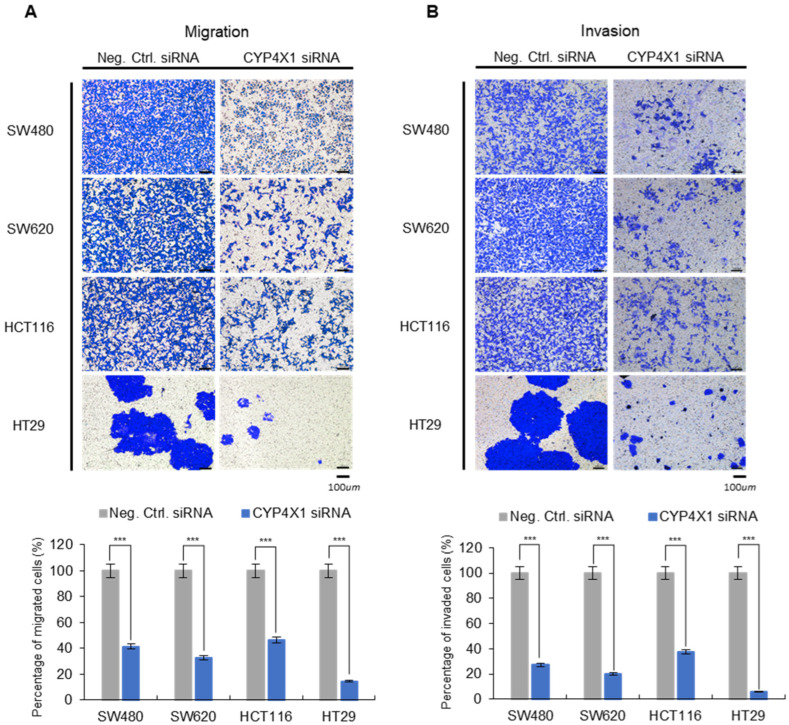
Transwell experiments demonstrated that *CYP4X1* downregulation suppressed the migration and invasion of colorectal cancer cells. (**A**) Image of cells that migrated to the lower chamber and quantification graph of cell migration (scale bar, 100 µm). (**B**) Image of cells that invaded the lower chamber and quantification graph of cell invasion (scale bar, 100 µm), (*** *p* < 0.001).

To investigate the effect of *CYP4X1* expression on the metastasis of CRC cells (SW480, SW620, HCT116, and HT29), its effects on cell migration and invasion were analyzed using a Transwell assay. *CYP4X1* downregulation was associated with a significantly reduced migration of CRC cells. *CYP4X1* siRNA-treated cells displayed decreased migration compared with the negative control cells: SW480, 58.4 ± 18.4%; SW620, 67.1 ± 6.7%; HCT116, 53.2 ± 12.2%; and HT29; 85.5 ± 6.7% (*p* < 0.001; Figure 5A). Similarly, the downregulation of *CYP4X1* expression was associated with decreased cell invasion. *CYP4X1*-downregulated cells demonstrated a decreased ability to invade the bottom chamber compared to the negative control cells: SW480, 72.7 ± 14.4%; SW620, 79.8 ± 7.5%; HCT116, 62.3 ± 13.2%; and HT29, 93.9 ± 4.5% (*p* < 0.001; Figure 5B). These results indicate that *CYP4X1* is significantly involved in cell migration and invasion.

### 2.6. CYP4X1 Knockdown Significantly Inhibits Colorectal Cancer Tumor Formation Both In Vitro and In Vivo

**Figure 6:**
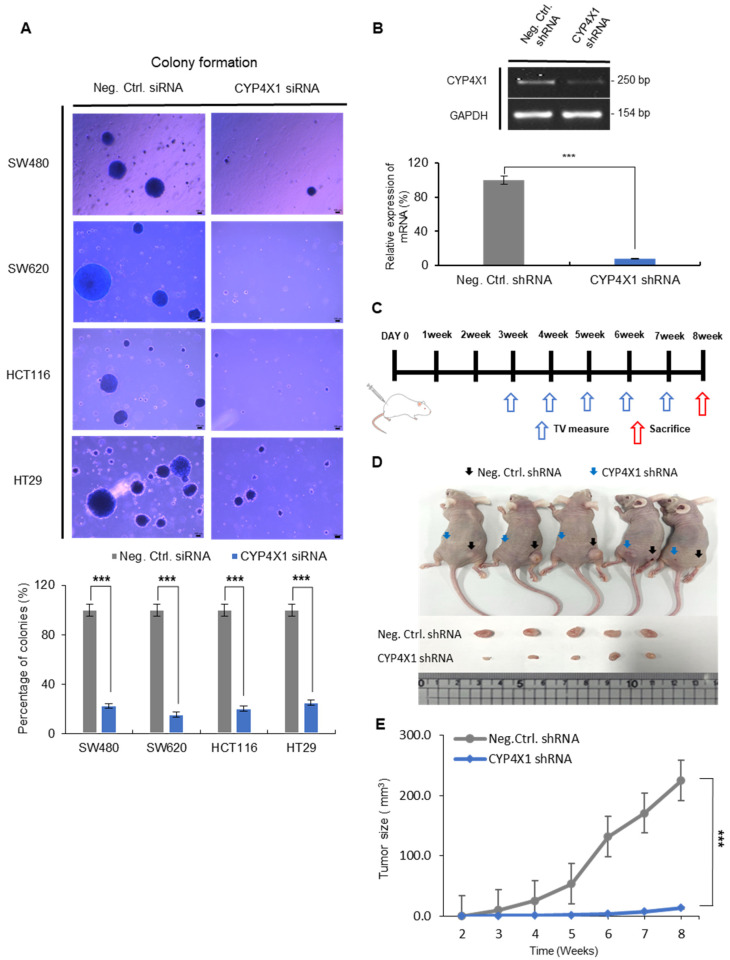
*CYP4X1* depletion reduces colony formation and tumor growth in CRC models. (**A**) Representative colony images and quantification of colony formation in soft agar after *CYP4X1* siRNA treatment (scale bar, 100 µm, *** *p* < 0.001). (**B**) *CYP4X1* suppression in HCT116 cells using shRNA is demonstrated by showing mRNA levels through PCR products. (**C**) CRC cells were injected into 6-week-old BALB/c nude mice, and tumor size was measured weekly starting from the 3rd week. (**D**) Images of xenograft tumors in mice with *CYP4X1* shRNA knockdown (left, blue arrow) and control (right, black arrow) after 8 weeks. (**E**) Tumor volume comparison between control and *CYP4X1* shRNA groups over time (*** *p* < 0.001).

The colony formation assay is a definitive indicator of tumorigenesis, as only cancerous cells exhibit anchorage-independent colony growth. To assess the effect of *CYP4X1* on tumorigenesis, we performed a soft agar colony formation assay using CRC cells. Cells with downregulated *CYP4X1* expression demonstrated a reduced ability to form colonies compared to the negative control: SW480, 77.7 ± 12.2%; SW620, 84.5 ± 9.7%; HCT116, 79.8 ± 13.3%; and HT29, 75.0 ± 14.0% (*p* < 0.001; Figure 6A). In addition, an in vivo analysis was conducted to elucidate the regulatory role of *CYP4X1* in colorectal cancer tumorigenesis. We used shRNA to knock down *CYP4X1* expression in HCT116 colorectal cancer cells (HCT116; 91.4% ± 2.8%, *p* < 0.001; Figure 6B). A xenograft mouse model was established by implanting both *CYP4X1*-expressing cells and *CYP4X1*-knockdown cells. *CYP4X1* knockdown (KD) HCT116 cells and control cells were subcutaneously injected into the left and right thighs of the same BALB/c nude mice, respectively, and tumor volumes were measured weekly from weeks 3 to 8 (Figure 6C). The *CYP4X1* KD HCT116 cells formed smaller tumor masses and exhibited slower tumor growth compared to the control cells (*p* < 0.001; Figure 6D,E). These findings suggest that *CYP4X1* knockdown significantly decreases CRC tumor formation.

## 3. Discussion

This study focused on elucidating the role of *CYP4X1* in colorectal cancer (CRC). Based on TCGA databases such as GENT2, UALCAN, and GEPIA, we analyzed *CYP4X1* expression at the mRNA level in both tumor and normal tissues and investigated its correlation with patient survival. The results show that *CYP4X1* expression was significantly higher in colon adenocarcinoma (COAD) and rectal adenocarcinoma (READ) compared to normal tissues, as further confirmed through UALCAN analysis. *CYP4X1* expression was found to be considerably elevated across various clinicopathological factors in normal and cancerous tissues. In our laboratory, protein-level analysis using immunohistochemical (IHC) staining of tissues from 243 CRC patients revealed that *CYP4X1* expression was consistently increased across multiple clinicopathological factors, including TNM stage, tumor differentiation, depth of invasion, and metastasis. Both mRNA and protein expression levels of *CYP4X1* were significantly higher in CRC tissues compared to normal colon tissues, consistent with the findings from the TCGA database and our laboratory experiments.

In particular, high *CYP4X1* expression was associated with various factors such as TNM stage, tumor differentiation, depth of invasion, lymph node metastasis, and distant metastasis, as shown through chi-square analysis, Mann–Whitney U/Kruskal–Wallis H test analysis, and Cox regression univariate/multivariate analysis. A strong correlation between *CYP4X1* expression in CRC tissues and these clinicopathological factors was observed. According to other researchers, *CYP4X1* protein expression is linked to increased tumor grade in breast cancer [18]. Moreover, Kaplan–Meier analysis indicated that *CYP4X1* expression significantly impacted the 5-year survival rate of CRC patients. Therefore, the overexpression of *CYP4X1* may serve as an independent prognostic marker for CRC.

*CYP4X1* encodes an orphan cytochrome P450 enzyme primarily expressed in the brain, aorta, and breast [12,13]. Recent studies suggest that *CYP4X1*, along with its paralog CYP4Z1, plays a role in carcinogenesis and inflammatory diseases through the arachidonic acid pathway [21,22]. The upregulation of epoxyeicosatrienoic acids (EETs), products of this pathway, activates the EGFR and PI3K/AKT/MAPK signaling pathways, promoting tumor formation and metastasis [23,24]. Conversely, the inhibition of CYP expression reduces activity in these pathways, decreasing tumor cell adhesion and metastasis. Our in vitro experiments further demonstrated that the downregulation of *CYP4X1* significantly inhibited cell proliferation, migration, invasion, and colony formation in CRC cell lines. The in vivo experiments confirmed that *CYP4X1* expression significantly affected the tumorigenic potential of CRC, highlighting the potential of *CYP4X1* as a therapeutic target.

In conclusion, this study highlights the pivotal role of *CYP4X1* in CRC progression. The overexpression of *CYP4X1* is closely associated with key factors such as TNM stage, tumor differentiation, depth of invasion, and lymph node metastasis, which influence CRC patient survival. *CYP4X1* could serve as both a prognostic marker and therapeutic target in CRC treatment. Additionally, further research is needed to investigate the role of *CYP4X1* in regulating EETs and its impact on EGFR phosphorylation and downstream signaling pathways. Finally, this study underscores the potential of *CYP4X1* as a target for future therapeutic strategies in CRC treatments.

## 4. Materials and Methods

### 4.1. Analysis of CYP4X1 Expression

The expression patterns of *CYP4X1* in normal and tumor tissues were analyzed using the Gene Expression of Normal and Tumor tissues 2 (GENT2) (http://gent2.appex.kr/gent2/, accessed on 27 June 2024), Gene Expression Profiling Interactive Analysis (GEPIA) (http://gepia.cancer-pku.cn/index.html, accessed on 27 June 2024), and The University of ALabama at Birmingham CANcer data analysis Portal (UALCAN) databases (http://ualcan.path.uab.edu/index.html, accessed on 27 June 2024). We also conducted a comparative analysis of the expression pattern of *CYP4X1* in colon cancer (COAD) using the UALCAN database, where we examined variations based on sample types and various clinicopathological factors, including race, sex, age, cancer clinical stage, weight, lymph node metastasis status, and TP53 mutations.

### 4.2. Human Colorectal Carcinoma and Normal Colon Specimens

Formalin-fixed and paraffin-embedded tissue microarray (TMA) slides containing tissues from 243 patients with colorectal cancer were purchased from Superbiochips (Seoul, Republic of Korea, https://www.tissue-array.com/, accessed on 14 July 2024). Patients with CRC included 133 men and 110 women (male: female ratio, 1:0.59), and the average age of the population was 63.2 years (range, 25–88 years). Clinicopathological data, including TNM classification and clinical stage, are presented in Table 5. The tumor stage was determined according to the TNM classification of the American Joint Committee on Cancer.

### 4.3. Immunohistochemistry (IHC)

Colorectal cancer TMA slides were deparaffinized three times with xylene and rehydrated stepwise using graded ethanol. Epitope retrieval was performed in citrate buffer (pH 6.0) in a microwave oven for 20 min. Endogenous peroxidase activity was inhibited using 0.3% hydrogen peroxide for 30 min at 23 °C. For blocking, cells were treated with 3% bovine serum albumin (BSA) and then washed with phosphate-buffered saline containing 0.1% Tween-20 (PBST). Subsequently, the TMA slides were incubated with anti-*CYP4X1* rabbit polyclonal antibody (1:500, MyBioSource, Vancouver, WA, Canada) overnight at 4 °C and then incubated with the secondary anti-rabbit antibody (1:200, Thermo Fisher Scientific, Waltham, MA, USA) for 4 h at room temperature. After washing, tissues were stained with 3,3′-diaminobenzidine tetrahydrochloride-chromogen (DAB), counterstained with Mayer’s hematoxylin, and then observed under a microscope. The negative control was stained using the same procedure, but the primary antibodies were omitted.

### 4.4. IHC Data Analysis

*CYP4X1* expression on TMA slides was evaluated by three observers (Chang-Jin Kim, Hyoung Jong Kwak, and Dongjun Jeong) and scored based on the staining intensity and percentage of positive cells. The scores depended on the percentage of positive cells: 0 (0–10%), 1 (11–30%), 2 (31–50%), or 3 (51–100%). Staining intensity was also scored according to the following criteria: 0 (no expression), 1 (mild expression), 2 (moderate expression), and 3 (strong expression). The final score was obtained by multiplying the score of the percentage of positive cells by the staining intensity score. The slides were classified into either the high-expression group (score ≥ 4) or the low-expression group (score < 4).

### 4.5. Cell Lines and Cell Culture Conditions

Human colorectal cancer cell lines (SW480, SW620, HCT116, and HT29) were obtained from the Korean Cell Line Bank (KCLB; Seoul, Republic of Korea). All the cell lines were maintained in Roswell Park Memorial Institute (RPMI) 1640 medium (Hyclone, Logan, UT, USA) supplemented with 10% heat-inactivated fetal bovine serum (FBS, Corning, Steuben County, NY, USA), 100 U penicillin, and 100 µg/mL of streptomycin (Corning), and were cultured in 5% CO_2_ at 37 °C. All human colorectal cancer cells had been purchased in the last 5 years. Cell identity was confirmed using short tandem repeat profiling of the KCLB.

### 4.6. Transfection of Small Interfering RNA (siRNA)

We purchased a pooled *CYP4X1* siRNA consisting of three pre-designed *CYP4X1* siRNAs (Bioneer, Daejeon, Republic of Korea) and downregulated the expression of *CYP4X1* in human CRC cell lines according to the manufacturer’s protocol. The four CRC cell lines were seeded in 6-well plates with RPMI 1640 medium containing 10% FBS and were cultured for 24 h to achieve a cell confluency of 50–60%. Then, the culture medium was discarded, and 30 nM siRNA was mixed with Lipofectamine RNAiMAX (Thermo Fisher Scientific, Waltham, MA, USA) in Opti-MEM (Thermo Fisher Scientific). The cells were transfected for 48 h. The *CYP4X1*-specific siRNA sequence targets were NM_001320289.1, NM_001320290.1, NM_178033.1, and XM_017000973.1. The negative control consisted of CRC cells transfected with a non-targeting siRNA (AccuTarget™ Negative Control siRNA, Bioneer, Daejeon, Republic of Korea).

### 4.7. Reverse Transcription–Polymerase Chain Reaction (RT-PCR)

Total RNA was extracted from the negative control and *CYP4X1*-downregulated cells using an RNA extraction kit (GENEALL, Seoul, Republic of Korea) following the manufacturer’s instructions. RNA purity and concentration were assessed by measuring the optical density using a microvolume spectrophotometer (Pultton, CA, USA). Further, 500 ng of quantified RNA was reverse-transcribed into cDNA using a HyperScript RT Master Mix Kit (GENEALL). RT-PCR was performed using a SYBR Green Real-Time PCR Master Mix Kit (TOYOBO, Osaka, Japan). The primers used for the qRT-PCR are listed in Table 6. The amplicons were loaded onto 2% agarose gels and stained using NeoGreen dye (Neoscience, Daejeon, Republic of Korea). Amplicons were visualized using a FluoroBox (Neoscience) nucleic acid gel imaging system, and mRNA expression levels were analyzed using ImageJ 1.53t software (National Institutes of Health). All analyses were performed in triplicate.

### 4.8. Western Blotting

The total cellular proteins of the negative control and *CYP4X1*-inhibited cells were lysed with PRO-PREP (iNtRON, Seongnam, Republic of Korea) and quantified using a BCA kit (Thermo Fisher Scientific). Subsequently, 30 μg of total protein was separated using 10% sodium dodecyl sulfate–polyacrylamide gel electrophoresis. The separated proteins were transferred onto polyvinylidene fluoride membranes (Millipore, Billerica, MA, USA). The membranes were blocked with 5% BSA for 1 h. Primary antibodies were added to the blocked membranes and incubated overnight at 4 °C. The primary antibodies used were anti-*CYP4X1* rabbit polyclonal antibody (1:1000; MyBioSource, Vancouver, Canada) and anti-β-actin mouse monoclonal antibody (1:1000; Santacruz, Santa Cruz, CA, USA). The next day, the membranes were incubated for 2 h after adding secondary antibodies, which included rabbit anti-human IgG Fc secondary antibody, HRP (dilution 1:2000; Thermo Fisher Scientific), and mouse anti-human IgG1 Fc secondary antibody, HRP (dilution 1:5000; Thermo Fisher Scientific). The membranes were then visualized using reagents for enhanced chemiluminescence (ECL, Thermo Fisher Scientific) and a Molecular Imager ChemiDoc XRS+ System (Bio-Rad Laboratories, Hercules, CA, USA). The acquired images were analyzed using ImageJ 1.53t software.

### 4.9. Cell Proliferation Assay (WST-1 Assay)

A cell proliferation assay kit (EZ-CYTOX, DAEILLAB SERVICE Co., Ltd., Seoul, Republic of Korea) was used to analyze the proliferation rate of CRC cells (SW480, SW620, HCT116, and HT29). Cells transfected with siRNA and negative control cells were harvested after 48 h, and 1 × 10^4^ cells per well were seeded onto a 96-well plate. The cells were then cultured at 37 °C with 5% CO_2_. At designated time points (24, 48, and 72 h), 5 mg/mL of EZ-Cytox Plus (DoGenBio, Seoul, Republic of Korea) was added to each well and incubated for 1 h at 37 °C. And the absorbance value was quantified at 450 nm using a microplate photometer. All analyses were performed in triplicate.

### 4.10. Migration and Invasion Assay (Transwell Assay)

Migration and invasion assays were performed using 6.5 mm Transwell inserts with 8.0 µm pore polycarbonate membranes. For the invasion assay, the inserts were coated with 1 mg/mL Matrigel (Corning), and the same procedure described for the migration assay was followed. Negative control cells and *CYP4X1*-siRNA-treated cells (5 × 10^5^ cells) were seeded onto the upper chamber in serum-free RPMI 1640 medium, while complete medium was added to the bottom chamber. The cells were incubated at 37 °C, 5% CO_2_ for 72 h. Cells from the upper chamber migrated (invaded) across the membrane to the bottom chamber. After 72 h, the cells remaining in the upper chamber were removed, and the membranes were washed three times with PBS. The cells that had migrated (invaded) to the bottom chamber were fixed with 3.7% formaldehyde (Biosesang, Seongnam, Republic of Korea) for 2 min. The cells were then treated with methanol for 20 min to increase permeability and were stained with 0.005% crystal violet for 20 min. All staining procedures were performed at 23 °C. The cells were counted under a microscope (Leica, Wetzlar, Germany) by randomly selecting five fields.

### 4.11. Soft Agar Colony Formation Assay

The bottom agar consisted of RPMI 1640 medium supplemented with 10% FBS and 0.5% agarose in a 6-well plate. The top agar, containing the cells, was composed of RPMI 1640 medium supplemented with 10% FBS and 0.35% agarose. After 48 h of siRNA treatment, 1 × 10^4^ cells per well, including negative control cells and *CYP4X1*-siRNA-treated cells, were seeded. After 21 days, the agarose was stained with 0.005% crystal violet, and five random fields were photographed. Colonies larger than 30 µm in diameter were counted.

### 4.12. Xenograft Tumorigenesis

To investigate the effect of *CYP4X1* on tumor cell growth in vivo, HCT116 cells were transfected with either the pGFP-C-shLenti vector (TR30021V, OriGene, Rockville, MD, USA) or *CYP4X1* human shRNA lentiviral particles (TL305119V, OriGene) to knock down *CYP4X1*. Six-week-old male BALB/c nude mice (OrientBio, https://www.orientbio.co.kr/ accessed on 14 July 2024) were bred at the Experimental Animal Center of Soonchunhyang Institute of Medi-Bio Science (SIMS). A total of 5 × 10^6^ transfected cells were subcutaneously inoculated into the mice, and tumor growth and volume were monitored weekly for 8 weeks. Tumor measurements were performed using digital calipers (Fowler Sylvac UltraCal Mark III; Sylvac, Lausanne, Switzerland). The average tumor volume was calculated using the following formula: tumor volume (TV; mm^3^) = W^2^ × L/2, where L represents the larger diameter and W represents the smaller diameter. All animal experiments were conducted in accordance with a protocol approved by the Institutional Animal Care and Use Committee of Soonchunhyang University (Approval No: SCH20-0071).

### 4.13. Statistical Analysis

All data are presented as the average of triplicate values. Statistical analysis was performed using SPSS v19 (SPSS Inc., Chicago, IL, USA), using an unpaired Student’s *t*-test. For the analysis of IHC scores, comparisons were made using the Mann–by Whitney U test, followed the Kruskal–Wallis test, and then the Dunn or Steel–Dwass post hoc tests. Chi-square analysis was used to determine the clinicopathological factors of patients with CRC according to *CYP4X1* expression. *CYP4X1* expression and the 95% risk ratio were assessed using Cox regression analysis, and the 5-year survival was assessed using Kaplan–Meier analysis between *CYP4X1* expression and patient outcome. A *p*-value < 0.05 was considered a statistically significant difference.

## Figures and Tables

**Table 1:** Performing UALCAN database analysis for COAD to investigate the differential expression of *CYP4X1* across various variables.

Variables	Different Stages	Comparisons	N	Statistical Significance
Sample types	Normal Primary tumor	Normal-Vs-Primary	41 286	<1 × 10^−12^
Individual cancer stages	Stage1 Stage2 Stage3 Stage4	Normal-Vs-Stage1 Normal-Vs-Stage2 Normal-Vs-Stage3 Normal-Vs-Stage4	45 110 80 39	1.79 × 10^−5^ 3.92 × 10^−10^ 5.54 × 10^−7^ 1.20 × 10^−3^
Patient’s race	Caucasian African American Asian	Normal-Vs-Caucasian Normal-Vs-African American Normal-Vs-Asian	193 55 11	1.95 × 10^−12^ 4.21 × 10^−6^ 2.78 × 10^−2^
Patient’s gender	Male Female	Normal-Vs-Male Normal-Vs-Female	156 127	1.43 × 10^−10^ 4.80 × 10^−11^
Patient’s weight ^1^	Normal weight Extreme weight Obese Extreme obese	Normal-Vs-Normal Weight Normal-Vs-Extreme Weight Normal-Vs-Obese Normal-Vs-Extreme Obese	70 74 56 10	1.54 × 10^−8^ 1.61 × 10^−5^ 4.80 × 10^−4^ 1.14 × 10^−1^
Patient’s age	21–40 y 41–60 y 61–80 y 81–100 y	Normal-Vs-Age(21–40y) Normal-Vs-Age(41–60y) Normal-Vs-Age(61–80y) Normal-Vs-Age(81–100y)	12 90 149 32	1.07 × 10^−1^ 4.78 × 10^−7^ 1.43 × 10^−11^ 6.58 × 10^−4^
Histological subtype	Adenocarcinoma Mucinous adenocarcinoma	Normal-Vs-Adenocarcinoma Normal-Vs-Mucinous -adenocarcinoma	243 37	1.11 × 10^−16^ 1.78 × 10^−4^
Nodal metastasis status ^2^	N0 N1 N2	Normal-Vs-N0 Normal-Vs-N1 Normal-Vs-N2	166 70 47	6.23 × 10^−14^ 3.39 × 10^−4^ 9.650 × 10^−5^
TP53 mutation status ^3^	TP53-Mutant TP53-NonMutant	Normal-Vs-TP53-Mutant Normal-Vs-TP53-NonMutant	160 122	1.13 × 10^−9^ 1.39 × 10^−14^

^1^ Normal weight, BMI greater than equal to 18.5 and BMI less than 25. Extreme weight, BMI greater than equal to 25 and BMI less than 30. Obese, BMI greater than equal to 30 and BMI less than 40. Extreme obese, BMI greater than 40. ^2^ N0, no regional lymph node metastasis; N1, metastases in 1 to 3 axillary lymph nodes; N2, metastases in 4 to 9 axillary lymph nodes; N3, metastases in 10 or more axillary lymph nodes. ^3^ TP53 mutation status was obtained from TCGA whole-exome sequencing data. We downloaded Mutation Annotation Format (MAF) files (derived from VarScan2) from the Genomic Data Commons portal. The samples with/without TP53 mutation were matched with RNA-seq data.

**Table 2:** Clinicopathological factors related to *CYP4X1* expression in patients with CRC.

Characteristics	*CYP4X1* Expression		Total	*p*-Value
	Low (*n* = 116)	High (*n* = 127)	(*n* = 243)	
Age, years, mean (SD)				
Gender, N (%)				0.397
Male	65 (48.9)	68 (51.1)	133	
Female	51 (46.4)	59 (53.6)	110	
pT, N (%)				0.138
T1 and T2	8 (34.8)	15 (65.2)	23	
T3 and T4	108 (49.1)	112 (50.9)	220	
pN, N (%)				<0.001
N0	86 (84.3)	16 (15.7)	102	
N1 and N2	30 (21.3)	111 (78.7)	141	
Metastasis, N (%)				0.407
M0	107 (48.2)	115 (51.8)	222	
M1	9 (42.9)	12 (57.1)	21	
Clinical stage, N (%)				<0.001
Stage I and Stage II	80 (83.3)	16 (16.7)	96	
Stage III and Stage IV	36 (24.5)	111 (75.5)	147	

**Table 3:** Results of Mann–Whitney U/Kruskal–Wallis H test analysis of the level of *CYP4X1* expression in CRC patients (*n* = 243).

Variables		N	U/H	*p*-Value
pN	pN0	102	1391.000	<0.001
	pN1-2	141		
Metastasis	M0	222	1458.500	0.004
	M1	21		
Clinical stage	Stage I	7	116.522	<0.001
	Stage II	89		
	Stage III	126		
	Stage IV	21		

**Table 4:** Univariate and multivariate analyses of clinicopathological factors in patients with CRC.

Characteristics	Variable	Univariate Analysis		Multivariate Analysis	
		Hazard Ratio (95% CI)	*p*-Value	Hazard Ratio (95% CI)	*p*-Value
Age	<60 year vs. ≥60 year	1.270 (0.858–1.879)	0.232	1.106 (0.741–1.650)	0.623
Gender	Male vs. Female	0.984 (0.669–1.448)	0.935	0.839 (0.564–1.248)	0.386
pT	T1, T2 vs. T3, T4	0.470 (0.271–0.813)	0.007	0.343 (0.191–0.618)	<0.001
pN	N0 vs. ≥N1, N2	11.833 (5.959–23.497)	<0.001	1.974 (0.488–7.985)	0.340
Metastasis	M0 vs. ≥M1	3.949 (2.364–6.597)	<0.001	4.097 (2.282–7.355)	<0.001
Clinical stage	Stage I, II vs. Stage III, IV	16.627 (7.277–37.988)	<0.001	3.959 (0.864–18.147)	0.076
*CYP4X1* expression	Low vs. High	8.068 (4.655–13.985)	<0.001	3.532 (1.813–6.880)	<0.001

**Table 5:** Information of tissues (TMA) used for immunohistochemistry staining.

Characteristic			N
Age			
	≤60		109
	>60		134
Sex			
	Male		133
	Female		110
pT			
	T1		0
	T2		23
	T3		187
	T4		33
pN			
	N0		102
	N1		96
	N2		45
Metastasis			
	M0		222
	M1		21
Clinical stage			
	Stage I		7
	Stage II		89
	Stage III		126
	Stage IV		21
Total			243

**Table 6:** Primers for RT-PCR.

Gene	Symbol;Acc	Reverse Primer Sequence	PCR Product
*CYP4X1* (siRNA)	HGNC:20244	F: 5′-TGAGCAGAACAGATCCCAAGT-3′	169 bp
		R: 5′-CAGAATGAGCCATCACCTCAAT-3′	
*CYP4X1* (shRNA)	HGNC:20244	F: 5′-TCAGCAAGCCACTTACCTTCCC-3′	250 bp
		R: 5′-TTCCAGACAGCAGGGTTGTGGT-3′	
*GAPDH*	HGNC:4141	F: 5′-GTCTCCTCTGACTCCAACAGCG-3′	154 bp
		R: 5′-ACCCTGTTGCTGTAGCCAA-3′	

## Data Availability

The datasets used and/or analyzed during the current study are available from the corresponding author on reasonable request.

## References

[1] Sung H., Ferlay J., Siegel R.L., Laversanne M., Soerjomataram I., Jemal A., Bray F. (2021). Global cancer statistics 2020: GLOBOCAN estimates of incidence and mortality worldwide for 36 cancers in 185 countries. CA Cancer J. Clin..

[2] Ryuk J.P., Choi G.-S., Park J.S., Kim H.J., Park S.Y., Yoon G.S., Jun S.H., Kwon Y.C. (2014). Predictive factors and the prognosis of recurrence of colorectal cancer within 2 years after curative resection. Ann. Surg. Treat. Res..

[3] Vatandoust S., Price T.J., Karapetis C.S. (2015). Colorectal cancer: Metastases to a single organ. World J. Gastroenterol..

[4] Siegel R.L., Miller K.D., Sauer A.G., Fedewa S.A., Butterly L.F., Anderson J.C., Cercek A., Smith R.A., Jemal A. (2020). Colorectal cancer statistics, 2020. CA Cancer J. Clin..

[5] Kumar S. (2015). Computational identification and binding analysis of orphan human cytochrome P450 4x1 enzyme with substrates. BMC Res. Notes..

[6] Danielson P.B. (2002). The cytochrome P450 superfamily: Biochemistry, evolution and drug metabolism in humans. Curr. Drug Metab..

[7] Nebert D.W., Russell D.W. (2002). Clinical importance of the cytochromes P450. Lancet.

[8] Guengerich F.P., Shimada T. (1998). Activation of procarcinogens by human cytochrome P450 enzymes. Mutat. Res..

[9] Windmill K.F., McKinnon R.A., Zhu X., Gaedigk A., Grant D.M., McManus M.E. (1997). The role of xenobiotic metabolizing enzymes in arylamine toxicity and carcinogenesis: Functional and localization studies. Mutat. Res..

[10] Kumarakulasingham M., Rooney P.H., Dundas S.R., Telfer C., Melvin W.T., Curran S., Murray G.I. (2005). Cytochrome P450 profile of colorectal cancer: Identification of markers of prognosis. Clin. Cancer Res..

[11] Sneha S., Baker S.C., Green A., Storr S., Aiyappa R., Martin S., Pors K. (2021). Intratumoural cytochrome P450 expression in breast cancer: Impact on standard of care treatment and new efforts to develop tumour-selective therapies. Biomedicines.

[12] Guengerich F.P., Cheng Q. (2011). Orphans in the human cytochrome P450 superfamily: Approaches to discovering functions and relevance in pharmacology. Pharmacol. Rev..

[13] Guengerich F.P., Wu Z.L., Bartleson C.J. (2005). Function of human cytochrome P450s: Characterization of the orphans. Biochem. Biophys. Res. Commun..

[14] Guengerich F.P., Tang Z., Salamanca-Pinzón S.G., Cheng Q. (2010). Characterizing proteins of unknown function: Orphan cytochrome P450 enzymes as a paradigm. Mol. Interv..

[15] Savas U., Hsu M.H., Griffin K.J., Bell D.R., Johnson E.F. (2005). Conditional regulation of the human *CYP4X1* and CYP4Z1 genes. Arch. Biochem. Biophys..

[16] Battista N., Fezza F., Maccarrone M. (2004). Endocannabinoids and their involvement in the neurovascular system. Curr. Neurovasc. Res..

[17] Choudhary D., Jansson I., Stoilov I., Sarfarazi M., Schenkman J.B. (2005). Expression patterns of mouse and human CYP orthologs (families 1–4) during development and in different adult tissues. Arch. Biochem. Biophys..

[18] Murray G.I., Patimalla S., Stewart K.N., Miller I.D., Heys S.D. (2010). Profiling the expression of cytochrome P450 in breast cancer. Histopathology.

[19] Zhang Y., Yuan Z., Shen R., Jiang Y., Xu W., Gu M., Gu X. (2020). Identification of biomarkers predicting the chemotherapeutic outcomes of capecitabine and oxaliplatin in patients with gastric cancer. Oncol. Lett..

[20] Bylund J., Zhang C., Harder D.R. (2002). Identification of a novel cytochrome P450, *CYP4X1*, with unique localization specific to the brain. Biochem. Biophys. Res. Commun..

[21] Capdevila J.H., Falck J.R. (2002). Biochemical and molecular properties of the cytochrome P450 arachidonic acid monooxygenases. Prostaglandins Other Lipid Mediat..

[22] Wang B., Wu L., Chen J., Dong L., Chen C., Wen Z., Hu J., Fleming I., Wang D.W. (2021). Metabolism pathways of arachidonic acids: Mechanisms and potential therapeutic targets. Signal Transduct. Target. Ther..

[23] Jiang J.G., Chen C.L., Card J.W., Yang S., Chen J.X., Fu X.N., Ning Y.G., Xiao X., Zeldin D.C., Wang D.W. (2005). Cytochrome P450 2J2 promotes the neoplastic phenotype of carcinoma cells and is up-regulated in human tumors. Cancer Res..

[24] Panigrahy D., Greene E.R., Pozzi A., Wang D.W., Zeldin D.C. (2011). EET signaling in cancer. Cancer Metastasis Rev..

